# Reduced Pain Medication Use Following Virtual Art Therapy in a Skilled Nursing Facility Patient: A Case Report

**DOI:** 10.7759/cureus.79652

**Published:** 2025-02-25

**Authors:** Mengdong He, Michelle Y Ko, Salim F Ahmed, Navid Darouian

**Affiliations:** 1 Medicine, University of California Los Angeles David Geffen School of Medicine, Los Angeles, USA; 2 Internal Medicine, University of California Los Angeles, Los Angeles, USA

**Keywords:** art therapy, chronic pain syndrome, insomnia, non-pharmacological therapy, skilled nursing facility care

## Abstract

Chronic pain and sleep disturbances significantly affect quality of life, particularly in older adults. Effective management often combines pharmacological and non-pharmacological interventions. Art therapy has shown benefits in reducing pain, promoting relaxation, and improving emotional well-being. While traditional art therapy is well-documented, virtual adaptations are less explored, especially in geriatric populations.

This report describes a 78-year-old woman with chronic pain and sleep disturbances linked to peripheral neuropathy, lower extremity vascular disease, and multiple musculoskeletal injuries. Despite treatment with gabapentin, oxycodone, and other medications, her sleep remained severely disrupted, averaging only one to 1.5 hours at a time. The patient began using “Vita Color for Seniors,” a virtual coloring application, dedicating one to 1.5 hours daily. Within two weeks, she reported sleeping four to five hours straight, a notable improvement, and reduced reliance on pain medication.

The structured and interactive design of the application likely facilitated meditative states, promoting relaxation and consistent use. Virtual art therapy may address physical and emotional challenges, offering an accessible intervention for older adults with chronic pain. This case highlights the potential of digital art therapy as a scalable adjunct to traditional treatments. Further research is needed to evaluate its long-term efficacy and broader applicability.

## Introduction

Chronic pain and associated sleep disturbances significantly impact quality of life, particularly in the geriatric population, with up to 37% of individuals with chronic pain being noted to have sleep disturbances [1]. Effective management often requires a multifaceted approach that includes pharmacological and non-pharmacological interventions. Among them, art therapy has emerged as a promising tool in addressing pain and sleep disorders due to its meditative and therapeutic effects [2,3]. Traditional art therapy involving physical media has been well-documented for its benefits in pain reduction and emotional well-being. Studies have shown that art therapy serves as an effective mind-body intervention, helping to distract individuals from pain, promote relaxation, and foster emotional expression. By enhancing coping mechanisms, it contributes to overall well-being and resilience [2]. Moreover, it offers a complementary approach that addresses both the physical and emotional aspects of chronic pain, which is particularly relevant for older adults with complex health needs [2,4].

Virtual art therapy, which uses digital platforms for artistic expression, has received less attention but shows significant potential, particularly for individuals with physical limitations. Structured and interactive art activities, as highlighted by Angheluta and Lee [4], engage patients in therapeutic processes that improve mood and reduce stress. Additionally, Luo et al. [3] emphasize the role of arts therapies in mitigating sleep initiation and maintenance disorders, a challenge commonly faced by older adults with chronic pain. Virtual art applications integrate artistic engagement, cognitive stimulation, and meditative practice, offering a novel and accessible intervention for geriatric populations. This case report explores the use of the virtual coloring book application "Vita Color for Seniors" to improve sleep quality and reduce dependence on pain medications in a 78-year-old woman with chronic pain and physical limitations. The findings underline the potential of digital art therapy as a cost-effective and impactful addition to traditional pain and sleep management strategies.

## Case presentation

A 78-year-old woman with a history of rheumatoid arthritis on chronic immunosuppressant therapy with multiple affected joints was admitted to a skilled nursing facility for rehabilitation after undergoing a right hip Girdlestone procedure. Over time, she transitioned to long-term care due to chronic pain and physical limitations that prevented independent living. Her chronic pain (rated from a seven to 10 on a scale of 10 daily), which had persisted for 10-15 years, was attributed to peripheral neuropathy, lower extremity peripheral artery disease, and multiple prior fractures and soft tissue injuries. These injuries included a full-thickness subscapularis tendon tear, bilateral tibial plateau fractures, left calcaneal fractures, and right tibial and fibular fractures. The pain severely disrupted her sleep, initially affecting sleep initiation and later causing frequent awakenings due to persistent foot pain, which significantly impaired restful sleep. Despite a regimen of gabapentin 300 mg three times a day, methocarbamol 1,000 mg three times a day, celecoxib 200 mg daily, oxycodone 5 mg every four hours, and melatonin 10 mg every evening, she managed to sleep only one to 1.5 hours at a time, profoundly impacting her quality of life.

The patient began using the “Vita Color for Seniors” virtual coloring application, a structured paint-by-numbers program designed to engage users in meditative artistic activities. She spent one to 1.5 hours daily on the activity and, within two weeks, reported improved sleep maintenance, sleeping four to five hours straight. This improvement was notable compared to her past experiences with traditional coloring books, which lacked the same structured and interactive elements. The virtual platform appeared to facilitate a meditative state, helping her achieve relaxation before sleep. Although she did not experience significant daytime pain relief, her improved nightly sleep duration indirectly reduced her reliance on as-needed pain medications, such as oxycodone, by decreasing their frequency of use. This highlights the potential adjunctive benefits of virtual art therapy.

## Discussion

This case underscores the potential of virtual art therapy as a novel tool for managing chronic pain and sleep disturbances in older adults. Studies have shown that art therapies, including virtual adaptations, can promote relaxation, enhance mood, and improve mental health outcomes. Raudenska et al. [2] and Angheluta and Lee [4] demonstrated that art therapy effectively reduces chronic pain, emphasizing its dual role in alleviating physical discomfort and addressing emotional challenges. Similarly, Luo et al. [3] highlighted art therapy's significant role in mitigating sleep initiation and maintenance disorders, which aligns with the improved sleep outcomes observed in this case. Additionally, Harasymchuk et al. [5] advocated for evidence-based approaches in arts-based chronic pain management, further supporting the structured design of virtual interventions.

Digital adaptations of art therapy, such as the "Vita Color for Seniors" application, remain underexplored but show promise for geriatric populations. The structured and interactive design of the application likely contributed to a meditative state, facilitating relaxation and improving sleep maintenance. Structured artistic activities, as highlighted by Angheluta and Lee [4], significantly enhance emotional and psychological well-being. Raudenska et al. [2] emphasized that engaging in creative and structured activities helps manage chronic pain and associated stress, making digital art therapy a scalable and impactful solution for older adults.

While the exact mechanisms remain unknown, the observed benefits may stem from several interconnected factors. First, the structured, paint-by-numbers design likely induced a flow state characterized by deep focus and reduced awareness of pain and stress. Second, the digital platform's convenience and accessibility encouraged consistent use, which is critical for therapeutic outcomes. Finally, the meditative nature of the activity promoted autonomic relaxation, enhancing sleep quality and reducing reliance on pain medications. This case demonstrates how virtual art therapy can engage older adults through accessible technological platforms, leveraging cognitive and sensory engagement to improve both emotional well-being and physical recovery.

## Conclusions

This case highlights the potential of virtual art therapy as an innovative and accessible intervention for chronic pain and sleep disturbances in older adults. By leveraging the therapeutic benefits of art, meditation, and technology, applications like “Vita Color for Seniors” can serve as valuable adjuncts to traditional pharmacological treatments. Future research should explore the broader applicability of digital art therapy in geriatric populations, examining its long-term efficacy, accessibility, and integration into holistic care models. Additionally, incorporating a qualitative scale could provide deeper insight into the impact of visual art therapy on chronic pain and sleep. This case underscores the importance of creative art therapy in the digital age, offering new methods for enhancing the quality of life in vulnerable populations.
